# eVLP-mediated CREM editing enhances cytotoxicity of bicistronic IL-15-armored CD19/BCMA dual-target CAR-NK cells

**DOI:** 10.3389/fimmu.2026.1976202

**Published:** 2026-10-01

**Authors:** Fengqin Shang, Guanlie Li, Shiting Gong, Lijiao Zhao, Bingyu Niu, Hanyi Chen, Yuchen Fu, Lijun Zhao, Jianxun Wang, Yuanyuan Shi

**Affiliations:** Shenzhen Cell Valley Biopharmaceuticals Group Co., Ltd., Shenzhen, China

**Keywords:** cancer immunotherapy, CAR-NK cells, CAR-T cells, CD19/BCMA dual targeting, CREM editing, engineered virus-like particles, genome editing, IL-15 armoring

## Abstract

Chimeric antigen receptor (CAR)-engineered natural killer (NK) cells represent a promising platform for cancer immunotherapy; however, optimization of CAR architecture and additional engineering strategies remain necessary to improve their functional activity. In this study, we generated CD19/BCMA dual-target CAR constructs based on tandem and bicistronic configurations with or without membrane-bound interleukin-15 (mbIL-15), and further evaluated engineered virus-like particle (eVLP)-mediated CREM editing as an additional strategy for CAR-NK cell engineering. Four CAR configurations were evaluated in primary human T and NK cells for CAR expression, cytotoxicity, cytokine production, and proliferative activity. Bicistronic CD19/BCMA CAR-mbIL-15 cells exhibited enhanced functional activity compared with corresponding tandem CAR-mbIL-15 cells, including increased tumor-cell killing and activation-associated functional responses *in vitro*. In a CD19/BCMA-positive leukemia xenograft model, bicistronic CD19/BCMA CAR-mbIL-15 T and NK cells reduced tumor burden, delayed disease progression, and prolonged survival. Furthermore, eVLP-mediated CREM editing achieved efficient genome modification in primary NK cells and remained compatible with subsequent CAR engineering. CREM-edited CAR-NK cells exhibited increased cytotoxic activity compared with unedited CAR-NK cells *in vitro*. Collectively, these findings support the development of modular CAR-NK engineering strategies integrating CAR architecture optimization, membrane-bound cytokine armoring, and transient genome engineering.

## Introduction

Chimeric antigen receptor (CAR)-modified immune cell therapies have achieved substantial clinical activity in hematological malignancies. CD19-targeted CAR-T cells can induce durable remissions in patients with relapsed or refractory B-cell malignancies, whereas B-cell maturation antigen (BCMA)-targeted CAR-T cells have demonstrated substantial clinical efficacy in multiple myeloma (1, 2). Nevertheless, relapse remains a major challenge following CAR-based therapies. Loss or alteration of target-antigen expression has been documented after both CD19- and BCMA-directed CAR-T therapy, including CD19 mutations and alternative splicing associated with antigen-negative relapse, as well as biallelic BCMA loss as a mechanism of resistance (3–5). These observations provide a rationale for developing multi-antigen targeting strategies to reduce dependence on a single tumor-associated antigen and improve therapeutic robustness.

Combined targeting of CD19 and BCMA has been clinically explored in multiple myeloma through sequential or combined administration of separately targeted CAR-T products, as well as through the use of dual-target CAR constructs (6–8). More broadly, multi-antigen CARs can be engineered using distinct receptor architectures. Tandem CARs incorporate two antigen-binding domains within a single receptor molecule, whereas bicistronic vectors enable co-expression of two independent CAR molecules. Direct comparisons of dual-target CAR formats have demonstrated that receptor configuration can influence antigen engagement, downstream signaling, and antitumor activity, while no single architecture appears to be universally optimal across different antigen-expression contexts (9, 10). These findings highlight the importance of empirically comparing tandem and bicistronic configurations during the development of dual-target CAR products.

CAR-NK cells represent an alternative cellular therapy platform with intrinsic CAR-independent cytotoxicity and potential compatibility with allogeneic manufacturing. Clinical studies of cord blood-derived CD19 CAR-NK cells incorporating IL-15 have demonstrated antitumor activity and provided evidence that CAR-NK cells can expand and persist after infusion (11, 12). IL-15 is a critical regulator of NK-cell survival, proliferation, and effector function, making cytokine armoring an attractive strategy for improving CAR-NK-cell activity. Expression of IL-15 in a membrane-bound form has been shown to support NK-cell growth and cytotoxicity (13), and more recent studies have extended this approach to CAR-NK cells, demonstrating that signaling-competent membrane-bound IL-15 can enhance their proliferative capacity and antitumor activity (14).

Cyclic AMP-responsive element modulator (CREM) has recently been implicated as a regulator of CAR-NK-cell function. CAR activation and IL-15 signaling can induce CREM expression, whereas CREM disruption has been reported to enhance CAR-NK-cell effector activity and modulate functional responses under tumor-associated suppressive conditions (15). These findings suggest that genome engineering may provide an additional strategy for optimizing IL-15-armored CAR-NK cells. Engineered virus-like particles (eVLPs) can deliver Cas9 or other genome-editing proteins as ribonucleoprotein cargo without requiring sustained expression of the editing machinery (16). Our group recently established a BaEV-enveloped eVLP platform for gene editing of immune cells and demonstrated its compatibility with sequential CAR engineering (17). Building on these observations, we generated tandem and bicistronic CD19/BCMA CARs with or without mbIL-15, compared the functional activity of mbIL-15-armored tandem and bicistronic configurations in primary T and NK cells, evaluated the selected bicistronic configuration *in vivo*, and subsequently examined whether eVLP-mediated CREM editing could be incorporated into this CAR-NK engineering workflow and evaluated its association with enhanced cytotoxic function.

## Methods

### Cell lines and culture

Phoenix-Ampho, BaEV-WT64, and HEK-293T cells used for retroviral vector production and titration were maintained in DMEM (Gibco, C11995500BT) supplemented with 10% fetal bovine serum (FBS; ExCell, FSP500) and 1% penicillin–streptomycin (P/S; Gibco, 15140-122) at 37 °C in 5% CO_2_.

NALM6-GFP-Luc cells were kindly provided by Prof. Yu Cao (Peking University) and were further engineered in our laboratory to express human BCMA, generating hBCMA-NALM6- GFP-Luc cells for *in vitro* cytotoxicity assays and the *in vivo* leukemia xenograft model. RPMI-GFP-Luc cells were kindly provided by Prof. Sen Wu (China Agricultural University), and hCD19-RPMI-GFP-Luc cells were previously generated in our laboratory for supplementary cytotoxicity assays. Raji cells were obtained from the American Type Culture Collection (ATCC) and were further engineered in our laboratory to generate Raji-GFP-Luc cells for supplementary cytotoxicity assays. All target cell lines were maintained in RPMI-1640 medium (Gibco, C11875500BT) supplemented with 10% FBS and 1% P/S at 37 °C in 5% CO_2_.

### CAR vector construction and retroviral production

Four CAR constructs were generated: Tandem CD19/BCMA CAR, Tandem CD19/BCMA CAR-mbIL-15, Bicistronic CD19/BCMA CAR, and Bicistronic CD19/BCMA CAR-mbIL-15. The tandem CAR incorporated CD19-VL, BCMA-VL, BCMA-VH, and CD19-VH within a single receptor containing a CD8 hinge/transmembrane domain, 4-1BB, and CD3ζ. The bicistronic construct co-expressed two independent CARs separated by a P2A peptide; the CD19 CAR contained CD28 hinge/transmembrane and costimulatory domains, whereas the BCMA CAR contained a CD8 hinge/transmembrane domain and 4-1BB. Membrane-bound IL-15 was introduced through an additional P2A-linked cassette. CAR sequences were synthesized by Tsingke Biotechnology, cloned into the corresponding retroviral vectors using the ClonExpress Ultra One Step Cloning Kit V2 (Vazyme, C116-01), and verified by Sanger sequencing.

Retroviral vectors were initially produced in Phoenix-Ampho cells and subsequently used to establish stable CAR-expressing BaEV-WT64 producer cells using the previously established BaEV-enveloped retroviral production platform (18). CAR expression was detected using APC-G4S (HyCells, GS-ARAP100) or FITC-labeled human BCMA/TNFRSF17 protein, Fc tag (ACRO Biosystems, BCA-HF254). Viral supernatants collected from BaEV-WT64 producer cells were pooled for primary T- and NK-cell transduction. Biological viral titers were determined in HEK-293T cells based on CAR-positive frequencies measured 48 h after transduction.

### Generation of primary CAR-T and CAR-NK cells

Human peripheral blood mononuclear cells (PBMCs) were obtained from three independent healthy donor-derived preparations provided by Miaoshun Biotechnology (catalog no. PB050C-W). The investigators were not involved in donor recruitment or sample collection.

For CAR-T-cell generation, thawed PBMCs were activated with OKT3 (100 ng/mL) and IL-2 (500 U/mL) in X-VIVO medium (Lonza, 04-418Q) supplemented with 10% FBS and 1% P/S. After 48 h, cells underwent two rounds of RetroNectin-assisted retroviral transduction using RetroNectin at 10 μg/mL and Polybrene at 6 μg/mL. CAR expression was assessed by flow cytometry 48–72 h after transduction. T-cell purity was quantitatively evaluated by flow cytometry based on the CD3^+^ population.

For CAR-NK-cell generation, primary NK cells were isolated from PBMCs using the MojoSort™ Human NK Cell Isolation Kit (BioLegend, 480054). NK-cell purity was quantitatively evaluated by flow cytometry. Product quality characterization was based on the CD3^-^CD56^+^ population, and representative purity values from three donor-derived CAR-NK cell preparations are summarized in **Supplementary Table S1**. Phenotypic analysis of CAR-NK cells used in functional experiments was further performed based on the CD3^-^CD16^+^CD56^+^ population. Purified NK cells were expanded in NK-cell complete medium (Shenzhen Cell Valley, SZ-CELL-RD5-004) and subjected to RetroNectin-assisted retroviral transduction after approximately 5 days of culture. CAR-positive frequencies were assessed approximately 72 h after transduction.

### Flow cytometry

CAR expression was detected using APC-G4S (HyCells, GS-ARAP100) or the FITC-labeled human BCMA-Fc protein (ACRO Biosystems, BCA-HF254). Corresponding untransduced T or NK cells were stained in parallel and used to define CAR-positive gates. Data were acquired using a CytoFLEX Flow Cytometer (Beckman Coulter, A00-1-1102) and analyzed using FlowJo™ v10.

### *In vitro* functional assays

Cytotoxicity was assessed by coculturing untransduced or CAR-engineered T and NK cells with hBCMA-NALM6-GFP-Luc cells for 16 h at effector-to-target (E:T) ratios of 1:16, 1:8, 1:4, 1:2, and 1:1. Target-cell viability was quantified by luciferase activity using a firefly luciferase reporter assay reagent (Beyotime, RG025M), and cytotoxicity was calculated as:


Cytotoxicity (%) = [1−luminescence of coculture wells/luminescence of target-only wells]×100%

For the supplementary cytotoxicity assay, hCD19-RPMI-GFP-Luc cells were cocultured for 16 h with untransduced NK cells or Bicistronic CD19/BCMA CAR-mbIL-15 NK cells at E:T ratios of 1:4, 1:2, 1:1, 2:1, and 4:1 using the same assay.

Antigen-induced Ki-67 expression was assessed after coculture with hBCMA-NALM6-GFP-Luc cells at an E:T ratio of 1:1 for 48 h in T-cell groups and 72 h in NK-cell groups. Cells were fixed, permeabilized, stained with APC-conjugated anti-Ki-67 antibody (BioLegend, 350513), and analyzed by flow cytometry.

For cytokine and effector molecule analysis, the indicated T- or NK-cell groups were cocultured with hBCMA-NALM6-GFP-Luc cells for 16 h at an E:T ratio of 1:4. TNF-α, IFN-γ, and granzyme B levels in culture supernatants were measured using the corresponding BioLegend assay kits (741192, 741195, and 741196).

### CREM expression and eVLP-mediated editing

CREM transcript levels were measured by RT-qPCR. Total RNA was extracted using the FastPure Cell/Tissue Total RNA Isolation Kit V2 (Vazyme, RC112-01) and reverse-transcribed using the HiScript III 1st Strand cDNA Synthesis Kit (+gDNA wiper) (Vazyme, R312-01). qPCR was performed using Taq Pro Universal SYBR qPCR Master Mix (Vazyme, Q712-02). Primer sequences were CREM-F, 5′-ACTGAATGAACTGTCCTCTGATG-3′; CREM-R, 5′-GTACTGCCATGGTAGCAATACT-3′; GAPDH-F, 5′-GAAGGTGAAGGTCGGAGT-3′; and GAPDH-R, 5′-GAAGATGGTGATGGGATTTC-3′. Relative CREM transcript levels were normalized to GAPDH and calculated using the comparative threshold cycle method (2^-ΔΔCt). The ΔCt value was calculated as Ct(CREM) − Ct(GAPDH), and ΔΔCt was calculated relative to the unedited NK-cell group, which was used as the calibrator (19).

CREM editing was performed using a BaEV-enveloped eVLP platform previously established by our group for gene editing of immune cells (17). The sgRNA targeted coding exon 4 of CREM (5′-ACCACCTAGTATTGCTACCA-3′; PAM, TGG). Primary NK cells were subjected to RetroNectin-assisted eVLP delivery on day 5 of culture in the presence of Polybrene (6 μg/mL) and were subsequently transduced with the Bicistronic CD19/BCMA CAR-mbIL-15 retroviral vector 48 h later. The CREM target locus was analyzed before and after CAR transduction by Sanger sequencing followed by ICE (Inference of CRISPR Edits) analysis to estimate indel frequencies (20). CREM-unedited and CREM-edited CAR-NK cells with comparable CAR-positive frequencies were used for cytotoxicity testing to minimize the potential impact of CAR expression differences.

### *In vivo* antileukemic efficacy

All animal procedures were reviewed and approved by the Institutional Animal Care and Use Committee of Shenzhen Rongwan Biotechnology Co., Ltd. (approval no. RW-IACUC-26-0053). Female M-NSG mice aged 6–8 weeks were obtained from Shanghai Model Organisms Center, Inc. and maintained under specific pathogen-free conditions at 22–23 °C with a 12-h light/dark cycle. Mice were intravenously inoculated with 2 × 10^5^ hBCMA-NALM6-GFP-Luc cells on day −4. Baseline bioluminescence imaging (BLI) was performed on day −1 using an AniView100 Pro MultiModel *In Vivo* Animal Imaging System following intraperitoneal administration of D-luciferin (PerkinElmer, 122799). Mice were stratified according to baseline BLI signal intensity and randomized into treatment groups (n = 6 per group).

On days 0 and 5, mice received intravenous infusions of untransduced T cells, untransduced NK cells, bicistronic CD19/BCMA CAR-mbIL-15 T cells, or bicistronic CD19/BCMA CAR-mbIL-15 NK cells. CAR-treated groups received 2 × 10^7^ total cells per mouse per infusion, corresponding to approximately 1 × 10^7^ CAR^+^ cells at a CAR-positive frequency of approximately 50%. Tumor burden was monitored by serial BLI on D2, D5, D8, D11, D14, D17, D22, and D27 after treatment initiation, and imaging data were analyzed using AniView Pro software. Body weight, clinical condition, and survival were monitored throughout the study. Animals reaching predefined humane endpoints were euthanized and included in the survival analysis.

### Peripheral blood analyses

Peripheral blood was collected 24 h after the first and second cell infusions (days 1 and 6). Following red blood cell lysis, genomic DNA was isolated from nucleated cells, and CAR transgene copies were quantified by probe-based qPCR targeting the CD19 CAR scFv region. Samples with signals below the lower limit of detection (LOD) were considered negative. Primers were CD19-VH-F, 5′-CCGGGCATCCCAGGAT-3′, and CD19-VH-R, 5′-TGCCGTCGGGCTTCTG-3′, with a VIC/MGB-labeled probe (5′-TTTCTAAGTATCTGAACTGGTACCA-3′).

Absolute quantification was performed using an external plasmid standard curve ranging from 10² to 10^7^ copies/μL. No endogenous reference gene was used; CAR transgene copies were normalized to the amount of gDNA analyzed and expressed as CAR copies/μg genomic DNA. Plasma collected at the same time points was analyzed for human IFN-γ as an indicator of systemic immune activation using the LEGENDplex™ Human CD8/NK Panel (BioLegend, 741189).

### Statistical analysis

Unless otherwise specified, *in vitro* experiments were performed using at least three independent donor-derived cell preparations. Animal sample sizes are indicated in the corresponding figure legends. Statistical analyses were performed using GraphPad Prism version 10.1.2. Quantitative data are presented as mean ± SD.

Comparisons between two independent groups were performed using two-tailed unpaired Welch’s *t*-tests. For comparisons involving multiple groups, ordinary one-way ANOVA followed by the indicated multiple-comparisons tests was used; Welch’s one-way ANOVA with appropriate heteroscedastic multiple-comparisons procedures was applied when variance heterogeneity was detected. Longitudinal BLI and body-weight data were analyzed using two-way repeated-measures ANOVA with Geisser–Greenhouse correction. For longitudinal BLI and body-weight analyses, only data collected from day −1 to day 22 were included in statistical comparisons. Data obtained at day 27 were presented descriptively for surviving animals and were not included in longitudinal analyses.

For overall cytotoxicity comparisons, the mean cytotoxicity across all tested effector-to-target (E:T) ratios was calculated for each independent experiment and used for statistical comparison between groups. Data spanning multiple orders of magnitude were log10-transformed before statistical analysis. Survival distributions were analyzed using the Kaplan–Meier method and compared using the two-sided log-rank (Mantel–Cox) test. Specific statistical tests and multiple-comparisons procedures are described in the corresponding figure legends. A two-sided *P* value < 0.05 was considered statistically significant.

## Results

### Expression of tandem and bicistronic CD19/BCMA CARs in primary T and NK cells

To compare alternative dual-target CAR architectures, we generated tandem and bicistronic CD19/BCMA CAR constructs, each with or without membrane-bound IL-15 (mbIL-15) (**Figure 1A**). In the tandem configuration, the CD19- and BCMA-binding domains were incorporated into a single CAR molecule, whereas the bicistronic construct co-expressed two independent CARs separated by a P2A peptide.

**Figure 1:**
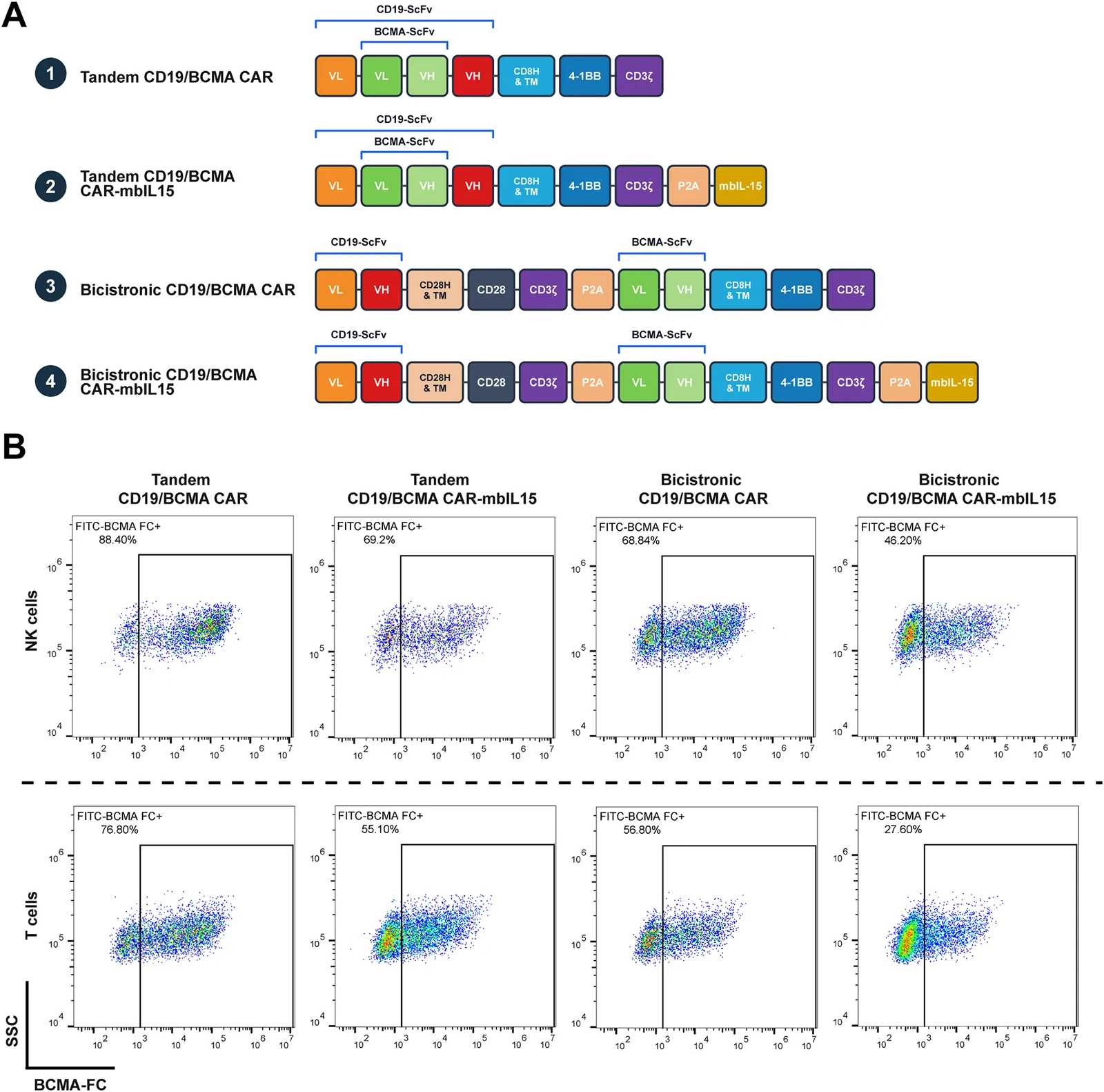
Design and expression of tandem and bicistronic CD19/BCMA CARs in primary T and NK cells. **(A)** Schematic representation of tandem and bicistronic CD19/BCMA CAR constructs with or without membrane-bound IL-15 (mbIL-15). In the tandem configuration, the CD19- and BCMA-targeting antigen-binding domains were incorporated into a single CAR, whereas the bicistronic construct co-expressed two independent CARs separated by a P2A peptide. **(B)** Representative flow cytometry plots showing surface CAR expression in primary T and NK cells transduced with the indicated constructs. CAR-positive populations were identified using FITC-labeled BCMA-Fc.

The four constructs were subsequently introduced into primary T and NK cells, and surface CAR expression was assessed by BCMA staining. Representative flow cytometry plots showed detectable CAR-positive populations for all four constructs in both cell types, with differences in CAR-positive frequencies among the individual configurations (**Figure 1B**). These cells were therefore used for subsequent functional comparisons of the tandem and bicistronic CAR architectures.

### Bicistronic CD19/BCMA CAR-mbIL-15 cells show enhanced *in vitro* antitumor activity

We next compared the *in vitro* antitumor activity of tandem and bicistronic CD19/BCMA CAR-mbIL-15 cells using hBCMA-NALM6-GFP-Luc cells as the primary target. Untransduced T or NK cells and the corresponding tandem or bicistronic CAR-mbIL-15 cells were cocultured with target cells across a range of E:T ratios, and target-cell killing was quantified using a luciferase-based assay (**Figures 2A–C**). Cytotoxicity increased with increasing E:T ratios in the CAR-engineered groups. When cytotoxicity was summarized across all five tested E:T ratios for each independent experiment, bicistronic CAR-mbIL-15 T cells and NK cells exhibited significantly greater overall tumor-cell killing than their respective untransduced controls and tandem CAR-mbIL-15 counterparts (**Figures 2B, C**).

**Figure 2:**
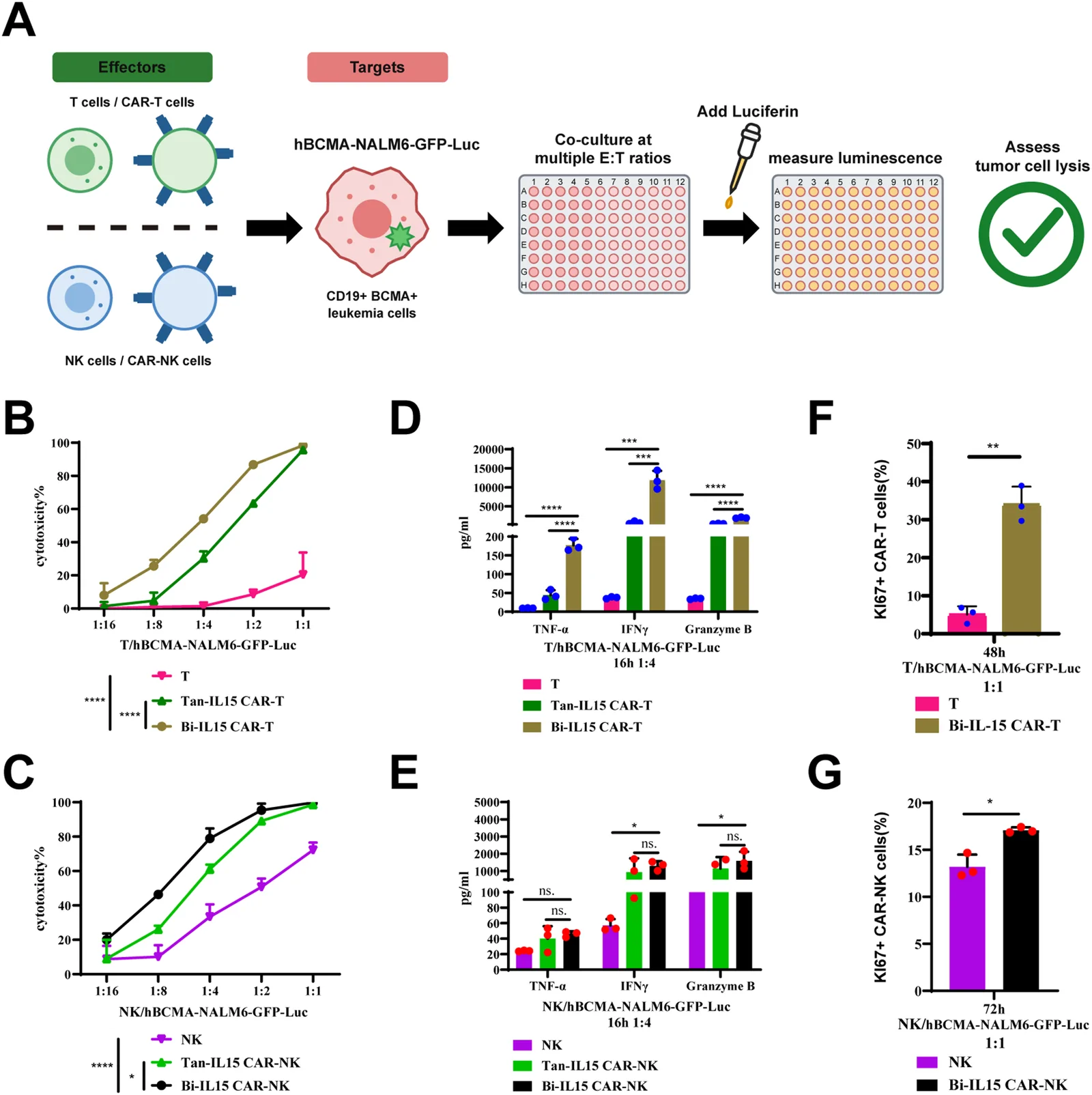
Bicistronic CD19/BCMA CAR-mbIL-15 enhances *in vitro* antitumor activity. **(A)** Schematic workflow of the luciferase-based cytotoxicity assay using hBCMA-NALM6-GFP-Luc target cells. Effector and target cells were cocultured at the indicated effector-to-target (E:T) ratios for 16 h before luminescence measurement. **(B, C)** Cytotoxicity of untransduced T cells, tandem CD19/BCMA CAR-mbIL-15 T cells (Tan-IL15 CAR-T), and bicistronic CD19/BCMA CAR-mbIL-15 T cells (Bi-IL15 CAR-T) **(B)**, or the corresponding NK-cell groups **(C)**, against hBCMA-NALM6-GFP-Luc cells at the indicated E:T ratios. **(D, E)** TNF-α, IFN-γ, and granzyme B levels in coculture supernatants from the indicated T-cell groups **(D)** and NK-cell groups **(E)** after 16 h of coculture with hBCMA-NALM6-GFP-Luc cells at an E:T ratio of 1:4. **(F)** Percentage of Ki-67^+^ cells in untransduced T cells and Bi-IL15 CAR-T cells after 48 h of coculture with hBCMA-NALM6-GFP-Luc cells at an E:T ratio of 1:1. **(G)** Percentage of Ki-67^+^ cells in untransduced NK cells and Bi-IL15 CAR-NK cells after 72 h of coculture with hBCMA-NALM6-GFP-Luc cells at an E:T ratio of 1:1. Data are presented as mean ± SD from three independent donor-derived experiments. For B–E, differences among groups were analyzed using ordinary one-way ANOVA followed by Dunnett’s multiple-comparisons test for the indicated comparisons. For B and C, the mean cytotoxicity across the five tested E:T ratios was calculated for each independent experiment before statistical analysis. For F and G, two-tailed unpaired Welch’s t-tests were used. *P < 0.05, **P < 0.01, ***P < 0.001, ****P < 0.0001; ns, not significant.

We next examined cytokine and effector molecule release following antigen coculture. Bicistronic CAR-mbIL-15 T cells produced significantly higher levels of TNF-α, IFN-γ, and granzyme B than both untransduced T cells and tandem CAR-mbIL-15 T cells (**Figure 2D**). In NK cells, bicistronic CAR-mbIL-15 cells showed significantly higher IFN-γ and granzyme B levels than untransduced NK cells, whereas TNF-α was not significantly different. No significant differences in TNF-α, IFN-γ, or granzyme B were detected between bicistronic and tandem CAR-mbIL-15 NK cells (**Figure 2E**).

Antigen-induced proliferation-associated responses were assessed by Ki-67 staining. Following coculture with hBCMA-NALM6-GFP-Luc cells, the proportion of Ki-67^+^ cells was significantly higher in bicistronic CAR-mbIL-15 T cells than in untransduced T cells at 48 h and was likewise increased in bicistronic CAR-mbIL-15 NK cells relative to untransduced NK cells at 72 h (**Figures 2F, G**; **Supplementary Figure S5**). These results indicate increased Ki-67 expression following antigen stimulation in both CAR-engineered cell types.

The bicistronic CAR-mbIL-15 NK-cell configuration was additionally evaluated against CD19/BCMA-positive hCD19-RPMI-GFP-Luc cells. Bicistronic CAR-mbIL-15 NK cells showed significantly greater overall tumor-cell killing than untransduced NK cells across the tested E:T ratios (**Supplementary Figure S3**).

Based on its overall cytotoxic activity and the associated functional responses observed under the conditions tested, the bicistronic CD19/BCMA CAR-mbIL-15 configuration was selected for subsequent *in vivo* evaluation.

To further evaluate the contribution of membrane-bound IL-15 expression to CAR-cell function, matched bicistronic CD19/BCMA CAR and CAR-mbIL-15 constructs were directly compared *in vitro*. Bicistronic CD19/BCMA CAR-mbIL-15 T cells and NK cells demonstrated enhanced cytotoxic activity compared with their corresponding non-armored CAR counterparts under the tested conditions (**Supplementary Figure S4**).

### Bicistronic CD19/BCMA CAR-mbIL-15 T and NK cells suppress leukemia progression *in vivo*

The *in vivo* antileukemic activity of bicistronic CD19/BCMA CAR-mbIL-15 T and NK cells was evaluated in an M-NSG xenograft model established with hBCMA-NALM6-GFP-Luc cells. Tumor-bearing mice received two infusions of untransduced T or NK cells or the corresponding bicistronic CAR-mbIL-15 cells on days 0 and 5, and leukemia burden was monitored longitudinally by bioluminescence imaging (**Figure 3A**).

**Figure 3:**
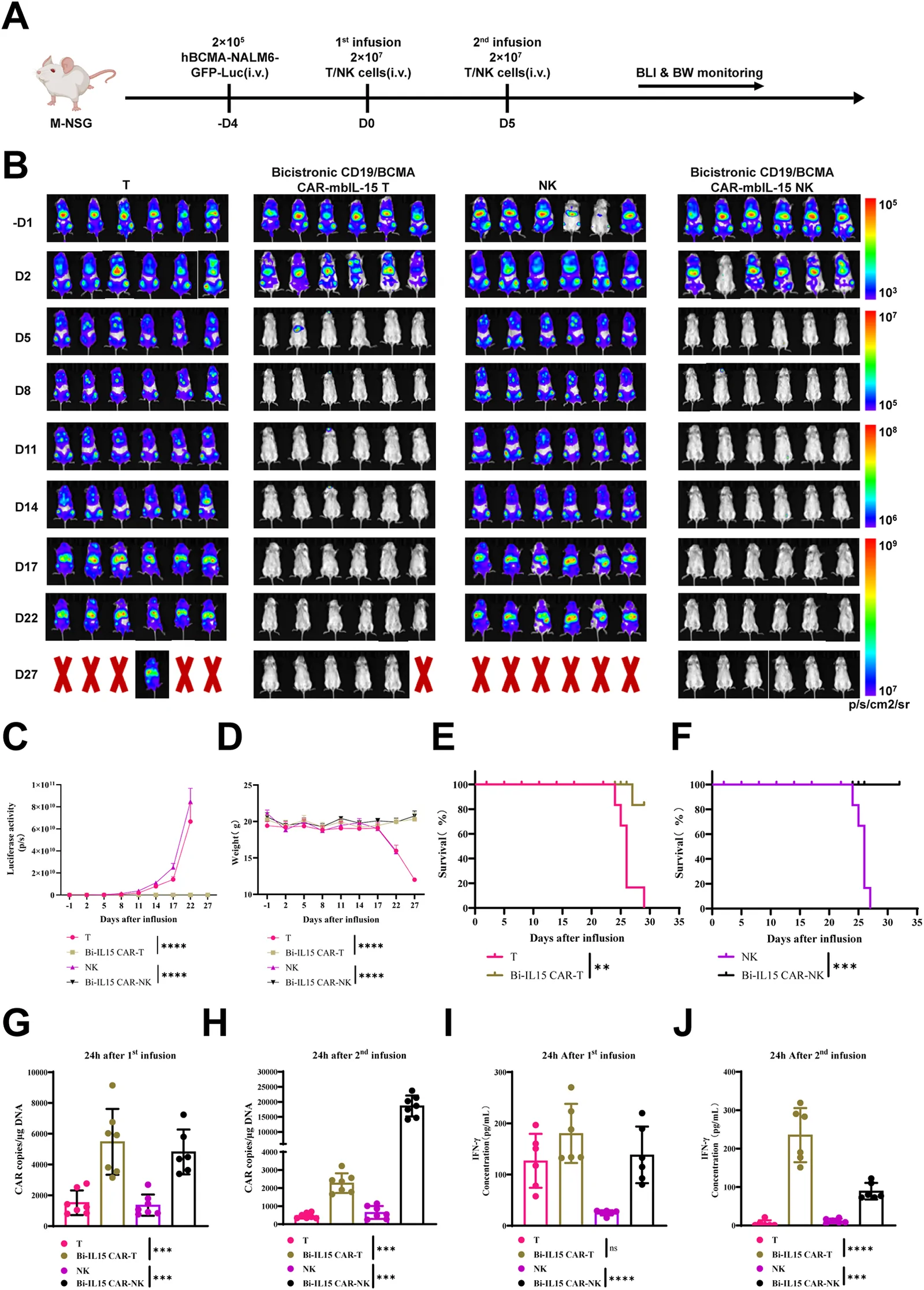
*In vivo* antileukemic activity of bicistronic CD19/BCMA CAR-mbIL-15 T and NK cells. **(A)** Experimental timeline of the hBCMA-NALM6-GFP-Luc xenograft study. M-NSG mice were intravenously inoculated with hBCMA-NALM6-GFP-Luc cells on day −4 and received two infusions of the indicated T- or NK-cell products on days 0 and 5. **(B)** Representative serial bioluminescence images showing leukemia burden during the observation period. Red crosses indicate animal death. **(C)** Quantification of total bioluminescence signals over time. **(D)** Changes in body weight during the observation period. **(E, F)** Kaplan–Meier survival curves for the T-cell cohort **(E)** and NK-cell cohort **(F)**. **(G, H)** Peripheral blood CAR transgene copy numbers measured 24 h after the first **(G)** and second **(H)** cell infusions. **(I, J)** Plasma IFN-γ levels measured as an indicator of systemic immune activation 24 h after the first **(I)** and second **(J)** cell infusions. Data are presented as mean ± SD, except for survival curves; n = 6 mice per group. For C and D, longitudinal BLI and body-weight data from D−1 to D22 were analyzed separately for T-cell and NK-cell cohorts using two-way repeated-measures ANOVA with Geisser–Greenhouse correction. The indicated significance corresponds to the treatment × time interaction. Data collected at D27 were presented descriptively for surviving animals and were not included in longitudinal statistical analyses. Bioluminescence data were log10-transformed before analysis. For E and F, survival curves were compared using the two-sided log-rank (Mantel–Cox) test. For G–J, log10-transformed data were analyzed using ordinary one-way ANOVA followed by Šídák’s multiple-comparisons test for the indicated comparisons. **P < 0.01, ***P < 0.001, ****P < 0.0001; ns, not significant.

Bioluminescence signals progressively increased in mice receiving untransduced T or NK cells. In contrast, mice treated with bicistronic CAR-mbIL-15 T or NK cells showed lower tumor-associated bioluminescence during follow-up, with significant differences in longitudinal tumor-burden trajectories relative to their respective untransduced controls (**Figures 3B, C**). Body weight declined during later disease progression in the untransduced control groups, whereas mice in both CAR-treated groups maintained comparatively stable body weights over the observation period. Longitudinal body-weight trajectories differed significantly between each CAR-treated group and its corresponding control (**Figure 3D**).

Kaplan–Meier analysis showed that bicistronic CAR-mbIL-15 T-cell treatment significantly prolonged survival compared with untransduced T-cell treatment. A significant survival benefit was likewise observed for bicistronic CAR-mbIL-15 NK cells relative to untransduced NK cells (**Figures 3E, F**).

CAR transgene copies were measured in peripheral blood 24 h after the first and second cell infusions. CAR transgene levels were significantly higher in both CAR-treated groups than in their respective untransduced controls at both sampling time points (**Figures 3G, H**). Plasma IFN-γ levels, as an indicator of systemic immune activation, were significantly higher in the CAR-NK group than in the untransduced NK-cell group after the first infusion, whereas no significant difference was detected between CAR-T and untransduced T-cell groups at this time point. After the second infusion, plasma IFN-γ levels, as an indicator of systemic immune activation, were significantly higher in both CAR-treated groups than in their respective controls (**Figures 3I, J**).

### eVLP-mediated CREM editing reduces CREM transcript levels and is associated with enhanced CAR-NK cytotoxicity

To evaluate CREM-targeted engineering in bicistronic CD19/BCMA CAR-mbIL-15 NK cells, primary NK cells were first subjected to eVLP-mediated CREM editing and subsequently transduced with the CAR retroviral vector (**Figures 4A, B**). Sanger sequencing followed by ICE analysis estimated a CREM indel frequency of 94% before CAR transduction and 84% after subsequent CAR transduction (**Supplementary Figure S6**). These measurements indicate that a substantial edited population remained detectable following the sequential editing and CAR-transduction procedure.

**Figure 4:**
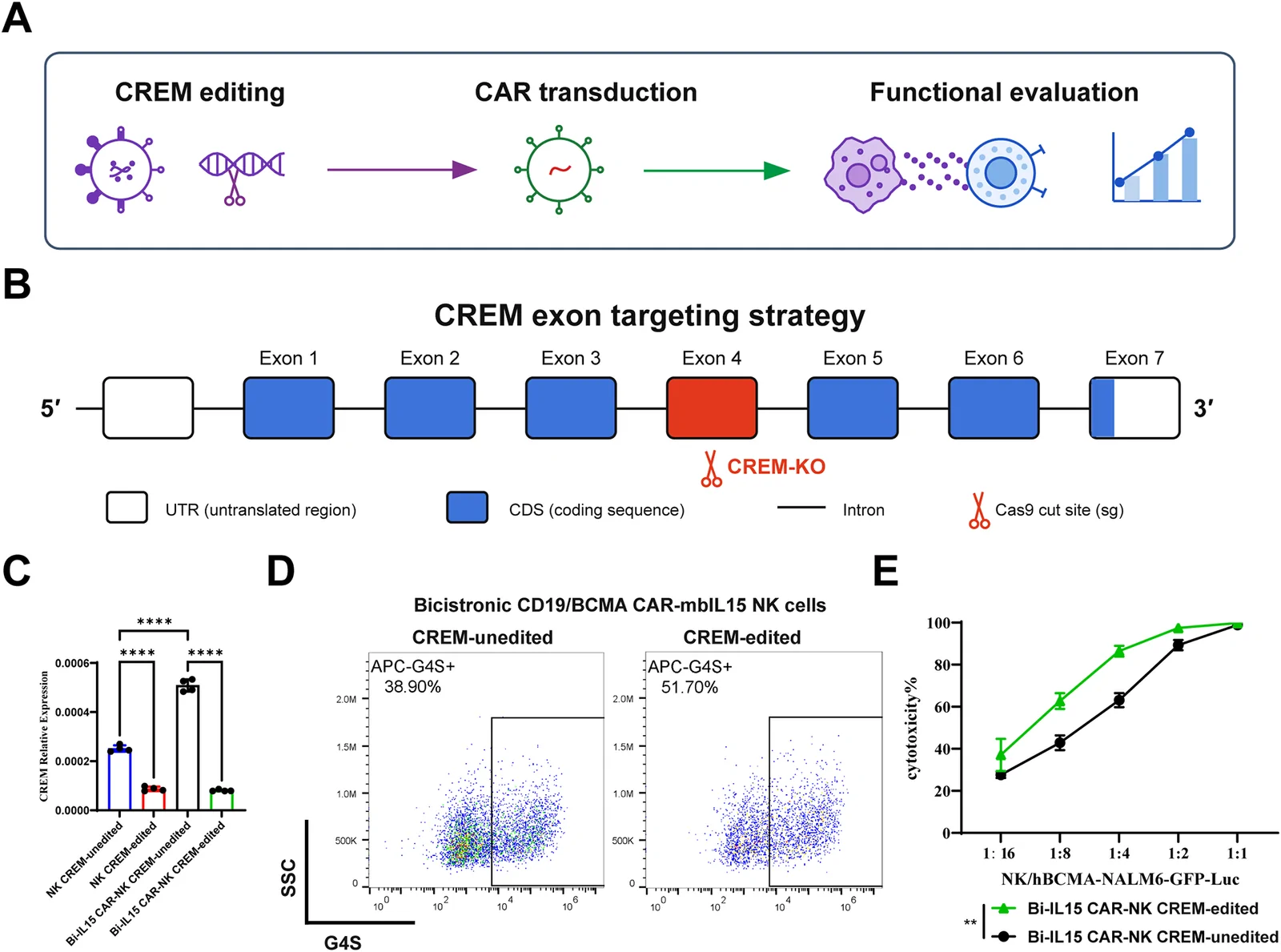
eVLP-mediated CREM editing reduces CREM transcript levels and enhances CAR-NK cytotoxicity. **(A)** Schematic workflow of eVLP-mediated CREM editing followed by bicistronic CD19/BCMA CAR-mbIL-15 transduction and functional evaluation. **(B)** Schematic representation of the CREM-targeting strategy. The sgRNA was designed to target coding exon 4 of CREM. **(C)** Relative CREM transcript levels in untransduced NK cells and bicistronic CD19/BCMA CAR-mbIL-15 NK cells with or without CREM editing, as determined by RT-qPCR (four independent cell samples derived from one donor). **(D)** Representative flow cytometry plots showing CAR-positive populations in CREM-unedited and CREM-edited bicistronic CD19/BCMA CAR-mbIL-15 NK cells. CAR-positive cells were detected by G4S staining. **(E)** Cytotoxicity of CREM-unedited and CREM-edited bicistronic CD19/BCMA CAR-mbIL-15 NK cells against hBCMA-NALM6-GFP-Luc cells at the indicated E:T ratios (three independent donor-derived experiments). CAR-positive frequencies were adjusted to comparable levels before the cytotoxicity assay. For **(C)**, data are presented as mean ± SD from four independent cell samples derived from one donor. Differences among groups were analyzed using Welch’s one-way ANOVA followed by Dunnett’s T3 multiple-comparisons test. For **(E)**, data are presented as mean ± SD from three independent donor-derived experiments. The mean cytotoxicity across all tested E:T ratios was calculated for each independent experiment, and the two groups were compared using a two-tailed unpaired Welch’s t-test. **P < 0.01, ****P < 0.0001; ns, not significant.

RT-qPCR analysis showed significantly higher CREM transcript levels in bicistronic CD19/BCMA CAR-mbIL-15 NK cells than in untransduced NK cells. CREM editing significantly reduced CREM transcript levels in NK cells, and CREM-edited bicistronic CAR-mbIL-15 NK cells likewise showed lower CREM transcript levels than their CREM-unedited counterparts (**Figure 4C**).

Representative flow cytometry showed CAR-positive populations in both CREM-unedited and CREM-edited bicistronic CAR-mbIL-15 NK cells, with representative CAR-positive frequencies of 38.9% and 51.7%, respectively (**Figure 4D**). These results indicate that CAR expression was retained after sequential CREM editing and CAR transduction. To evaluate the functional effect of CREM editing independently of differences in CAR-positive cell frequency, CREM-unedited and CREM-edited CAR-NK cells were adjusted to comparable CAR-positive frequencies before cytotoxicity testing. CREM-edited CAR-NK cells showed greater tumor-cell killing across several tested E:T ratios. When cytotoxicity was summarized across all tested E:T ratios for each independent experiment, CREM-edited CAR-NK cells exhibited significantly greater overall tumor-cell killing than CREM-unedited CAR-NK cells (**Figure 4E**).

## Discussion

This study evaluated an integrated engineering strategy combining CD19/BCMA dual targeting, membrane-bound IL-15 (mbIL-15) armoring, and CREM-directed genome editing in primary CAR-NK cells. Among the configurations examined, the bicistronic CD19/BCMA CAR-mbIL-15 construct showed a favorable *in vitro* functional profile and mediated antileukemic activity in the hBCMA-NALM6 xenograft model. Building on this configuration, eVLP-mediated CREM editing reduced CREM transcript levels and increased CAR-NK-cell cytotoxicity after edited and unedited cells were adjusted to comparable CAR-positive frequencies. These findings support further evaluation of CREM editing as an additional engineering strategy for IL-15-armored dual-target CAR-NK cells.

The functional consequences of multi-antigen targeting depend not only on antigen selection but also on CAR architecture. Bispecific and dual-CAR strategies have been developed to broaden antigen recognition and reduce vulnerability to antigen escape (21). Bicistronic dual-target CAR products have also entered clinical evaluation (22). Importantly, direct comparisons of tandem, bicistronic, and other multi-CAR configurations have shown that receptor organization can influence antigen binding, signaling, and functional activity, without establishing a universally optimal architecture across target combinations (9, 10). Antigen density may also dynamically influence CAR-cell activity and tumor escape, further emphasizing the importance of evaluating receptor design within the intended antigen context (23). In the present study, bicistronic CD19/BCMA CAR-mbIL-15 T and NK cells exhibited greater overall cytotoxicity than their tandem counterparts. However, these effects were not uniform across all functional readouts: bicistronic CAR-T cells showed increased TNF-α, IFN-γ, and granzyme B production, whereas no significant differences in these molecules were detected between tandem and bicistronic CAR-NK cells. Therefore, the current findings support a favorable functional profile of the bicistronic configuration under the conditions tested rather than universal superiority over tandem CAR designs. Moreover, only the bicistronic construct was evaluated *in vivo*, and the study did not directly examine single-antigen-loss or antigen-heterogeneous tumor models; therefore, *in vivo* superiority or protection against antigen escape cannot be concluded from the present data.

IL-15 armoring represents another strategy for improving NK-cell survival and functional activity. Clinical studies of CD19 CAR-NK cells incorporating IL-15 have demonstrated antitumor activity and provided evidence that engineered NK cells can expand and persist after infusion (11, 12). Preclinical studies have further shown that membrane-bound IL-15 can support NK-cell proliferation and cytotoxic function, including in CAR-NK platforms (13, 14). In the present study, bicistronic CD19/BCMA CAR-mbIL-15 T and NK cells showed increased Ki-67 expression following antigen stimulation, and the corresponding CAR-treated groups suppressed leukemia-associated bioluminescence and prolonged survival *in vivo*. CAR transgene copies were detectable in peripheral blood 24 h after both cell infusions, together with measurable plasma IFN-γ responses. These early measurements are consistent with the presence and functional engagement of infused CAR cells at the sampled time points but do not establish long-term expansion or persistence. However, plasma IFN-γ levels represent systemic immune activation and cannot be attributed specifically to CAR-NK cells. Furthermore, because a fully matched *in vivo* comparison between bicistronic CAR constructs with and without mbIL-15 was not performed, the independent contribution of mbIL-15 to the observed *in vivo* effects cannot be determined from the present experiments.

CREM editing provides a mechanistically distinct layer of engineering beyond CAR architecture and cytokine armoring. Rafei et al. identified CREM as a regulatory factor induced by CAR activation and IL-15 signaling in NK cells and demonstrated that CREM disruption enhanced CAR-NK-cell effector function *in vitro* and *in vivo* (15). More broadly, efficient CRISPR-mediated modification of primary human NK cells has been demonstrated for inhibitory or regulatory genes, and combined CAR engineering with intracellular regulatory-gene editing, including CISH disruption, has been shown to enhance CAR-NK-cell cytotoxicity (24, 25). eVLPs provide an alternative approach for delivering genome-editing ribonucleoproteins without requiring sustained expression of the editing machinery (16), and our previously established BaEV-enveloped eVLP platform demonstrated compatibility between eVLP-mediated editing and subsequent CAR engineering (17). In the present study, ICE analysis estimated CREM indel frequencies of 94% before and 84% after subsequent CAR transduction, while RT-qPCR demonstrated reduced CREM transcript levels in edited NK and CAR-NK cells. Because representative CAR-positive frequencies differed between edited and unedited preparations, the two populations were adjusted to comparable CAR-positive frequencies before functional testing. Under these conditions, CREM-edited CAR-NK cells exhibited significantly greater overall tumor-cell killing than their unedited counterparts, indicating that the observed functional difference was not attributable solely to differences in CAR-positive cell frequency. The retained estimated indel frequency after CAR transduction further supports the feasibility of incorporating eVLP-mediated CREM editing upstream of CAR engineering under the conditions tested. However, the current data do not establish complete CREM knockout, manufacturing robustness, or scalability of the combined CREM-editing/CAR-NK process.

Several limitations should be considered when interpreting these findings. First, although bicistronic CD19/BCMA CAR-mbIL-15 cells showed antitumor activity against CD19/BCMA co-expressing target cells, functional dissection using antigen-specific single-positive or antigen-negative target models was not performed. Therefore, the independent contribution of the CD19- and BCMA-targeting CAR arms, as well as the potential impact of antigen loss or heterogeneity, requires further investigation. Second, CREM-edited CAR-NK cells were evaluated functionally only *in vitro*; therefore, whether CREM editing provides additional tumor control, survival benefit, or altered cellular kinetics *in vivo* remains unknown. Second, although the *in vitro* experiments were performed using cells from three independent healthy donor-derived preparations, broader reproducibility across additional donors and manufacturing batches remains to be established. Third, although CREM editing was confirmed at the genomic level by Sanger sequencing/ICE analysis and was accompanied by reduced CREM transcript levels, CREM protein loss and the molecular mechanisms underlying enhanced cytotoxicity were not further investigated. In addition, comprehensive off-target and genomic-safety analyses were beyond the scope of this study. Future studies incorporating multiple donors and manufacturing runs, protein-level and genomic-safety characterization, antigen-heterogeneous tumor models, and *in vivo* evaluation of CREM-edited CAR-NK cells will be required to further define the reproducibility and translational relevance of this engineering strategy.

## Conclusion

In conclusion, we developed a modular CAR-NK engineering strategy integrating CD19/BCMA dual-targeting, membrane-bound IL-15 armoring, and eVLP-mediated CREM editing. The bicistronic CD19/BCMA CAR-mbIL-15 configuration demonstrated enhanced functional activity *in vitro* and antileukemic activity *in vivo*, while CREM editing was associated with enhanced CAR-NK cytotoxicity under the tested conditions. These findings support further optimization of engineered CAR-NK platforms through combined receptor, cytokine, and genome-engineering approaches.

## Data Availability

The original contributions presented in the study are included in the article/**Supplementary Material**. Further inquiries can be directed to the corresponding authors.
