# Supplementary material for: eVLP-mediated CREM editing enhances cytotoxicity of bicistronic IL-15-armored CD19/BCMA dual-target CAR-NK cells
Source: Front Immunol. 2026 Oct 1;17:1976202. doi: 10.3389/fimmu.2026.1976202 (PMC13668854; doi:10.3389/fimmu.2026.1976202)
Supplement: Supplementary file 1 [file DataSheet1.pdf]

Supplementary Figure Legends

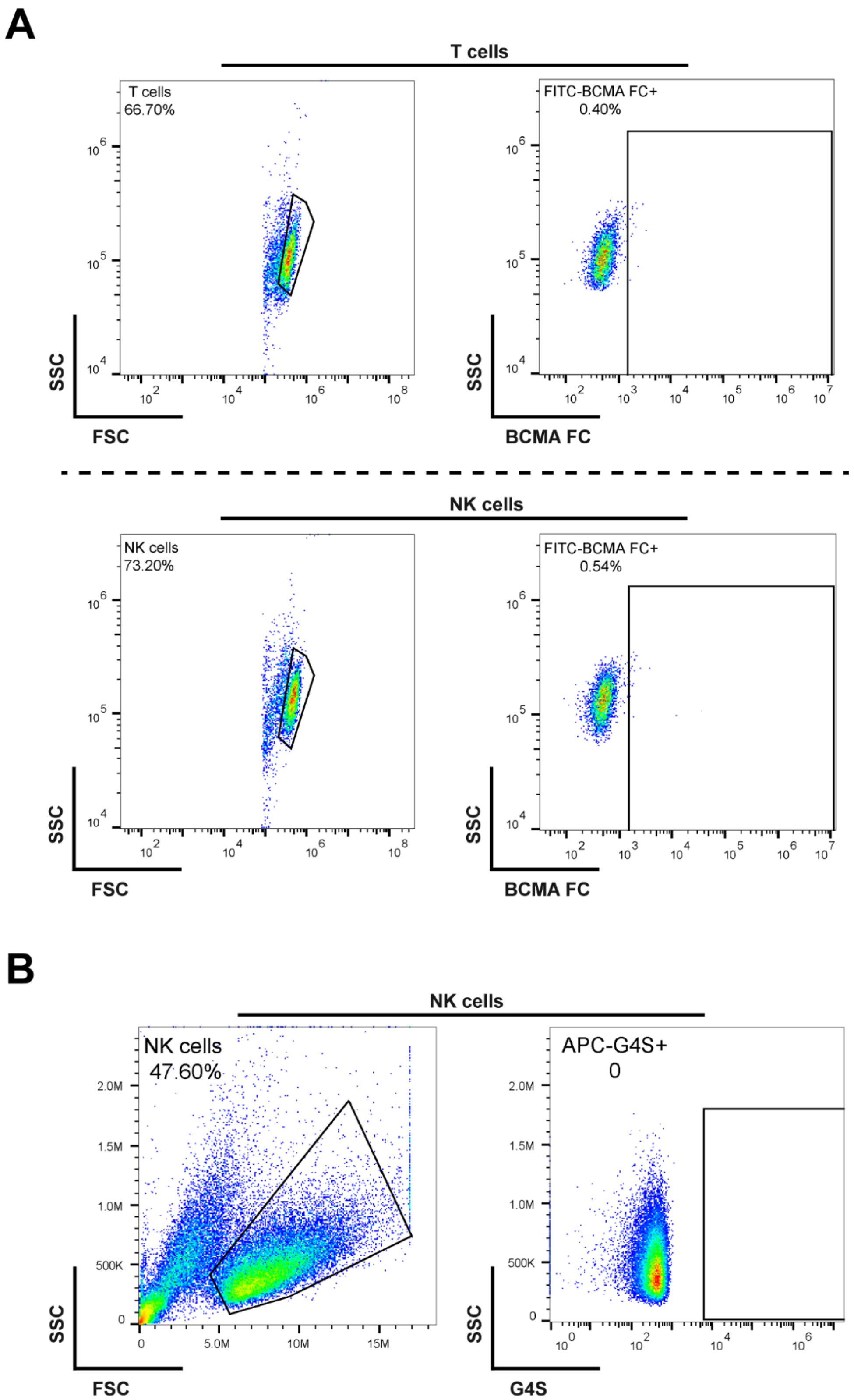

Supplementary Figure S1.

Representative negative controls for flow cytometric detection of CAR expression.

(A) Representative negative controls for FITC-labeled BCMA-Fc-based detection of CAR expression in untransduced T cells and NK cells. Untransduced T cells and NK cells stained in parallel were used to define CAR-positive gates.

(B) Representative negative controls for G4S-based detection of CAR expression in bicistronic CD19/BCMA CAR-mbIL-15 NK cells. Untransduced NK cells stained in parallel were used to define the CAR-positive gate.

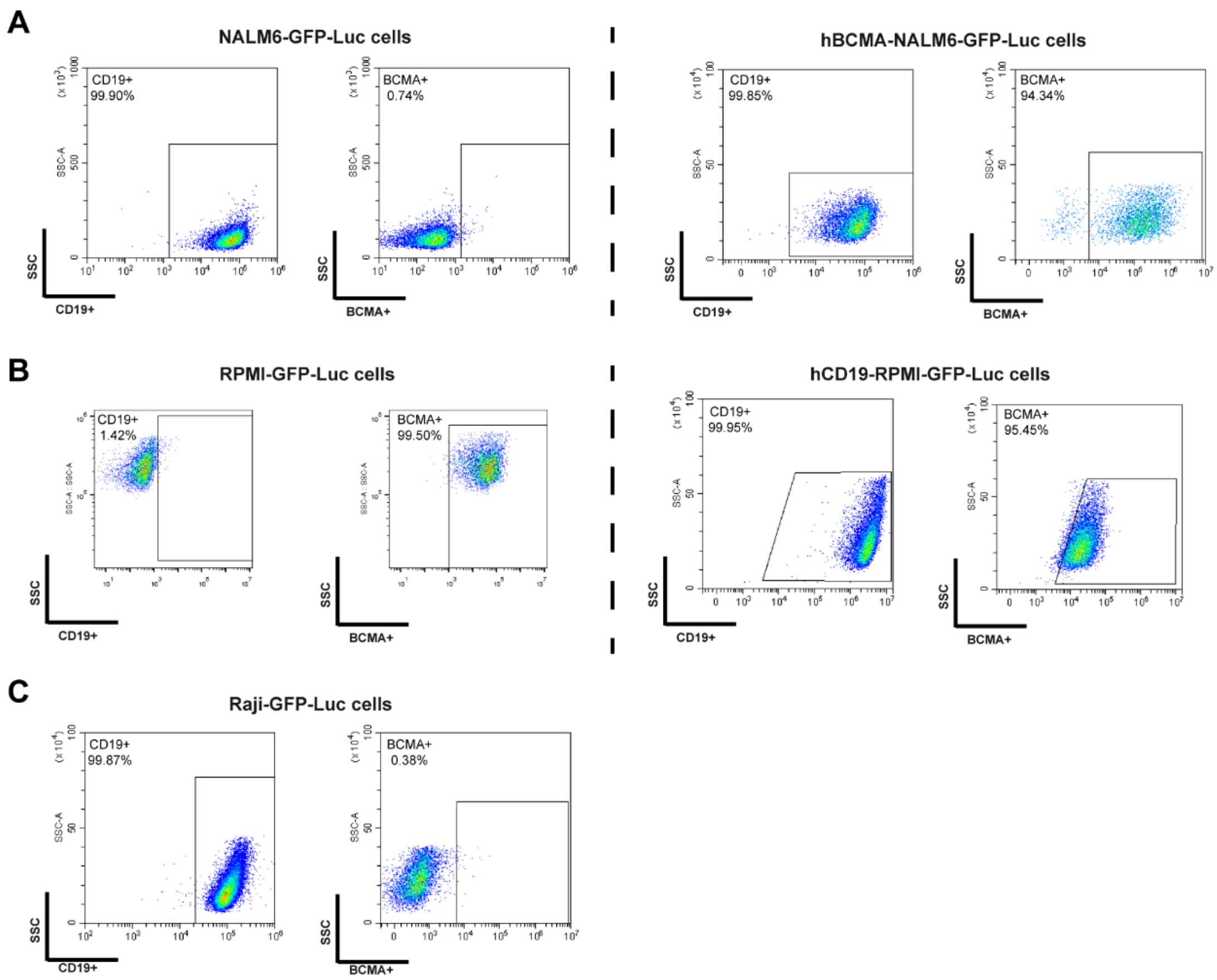

Supplementary Figure S2.

Characterization of CD19 and BCMA expression on target cell lines used for CAR-NK functional evaluation.

(A) Flow cytometric analysis of CD19 and BCMA expression on NALM6-GFP-Luc and hBCMA-NALM6-GFP-Luc target cells.

(B) Flow cytometric analysis of CD19 and BCMA expression on RPMI-GFP-Luc and hCD19-RPMI-GFP-Luc target cells.

(C) Flow cytometric analysis of CD19 and BCMA expression on Raji-GFP-Luc target cells.

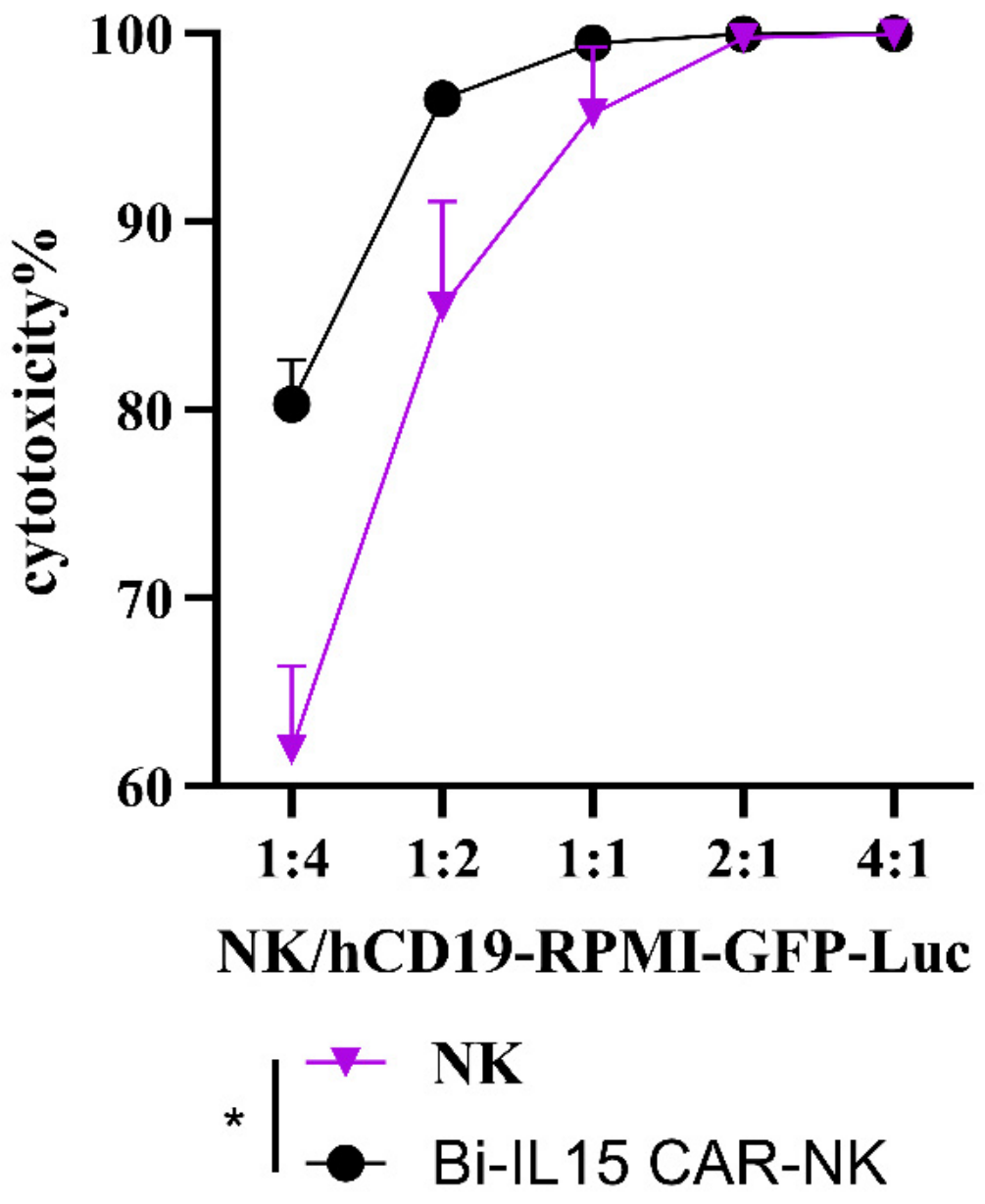

Supplementary Figure S3.

In vitro cytotoxicity of bicistronic CD19/BCMA CAR-mbIL-15 NK cells against CD19/BCMA-positive hCD19-RPMI-GFP-Luc target cells.

Cytotoxicity of untransduced NK cells and bicistronic CD19/BCMA CAR-mbIL-15 NK cells against CD19/BCMA-positive hCD19-RPMI-GFP-Luc target cells after 16 h of coculture at indicated effector-to-target (E:T) ratios.

Data are presented as mean ± SD from three independent healthy donor-derived cell preparations. Statistical analysis was performed using the methods described in the main manuscript.

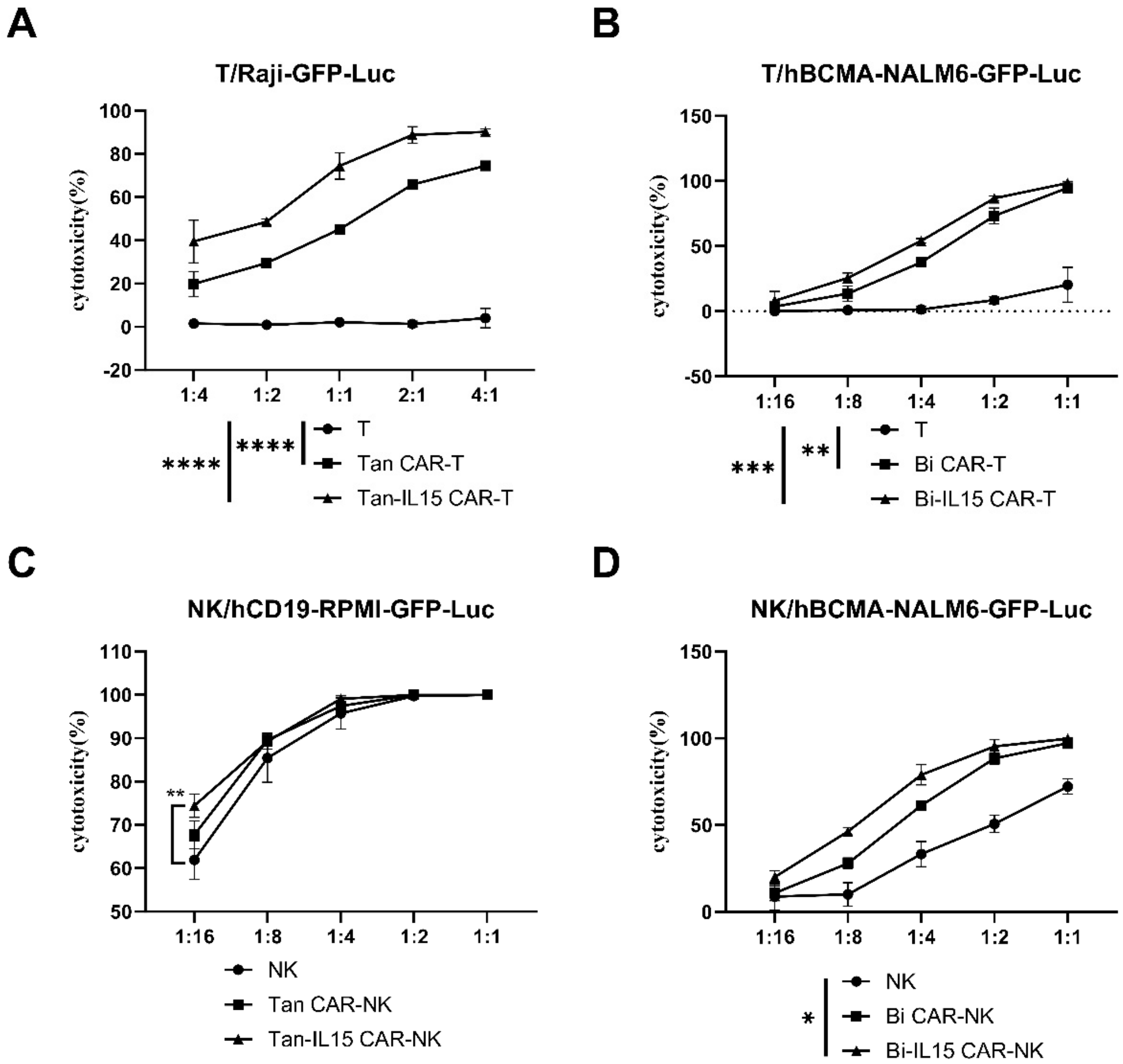

Supplementary Figure S4.

Effect of membrane-bound IL-15 expression on cytotoxic activity of CAR-engineered immune cells.

(A) Cytotoxicity of bicistronic CD19/BCMA CAR-T cells and bicistronic CD19/BCMA CAR-mbIL-15 T cells against Raji-GFP-Luc target cells at indicated effector-to-target (E:T) ratios.

(B) Cytotoxicity of bicistronic CD19/BCMA CAR-T cells and bicistronic CD19/BCMA CAR-mbIL-15 T cells against hBCMA-NALM6-GFP-Luc target cells at indicated effector-to-target (E:T) ratios.

(C) Cytotoxicity of untransduced NK cells, bicistronic CD19/BCMA CAR-NK cells, and bicistronic CD19/BCMA CAR-mbIL-15 NK cells against hCD19-RPMI-GFP-Luc target cells at indicated effector-to-target (E:T) ratios.

(D) Cytotoxicity of untransduced NK cells, bicistronic CD19/BCMA CAR-NK cells, and bicistronic CD19/BCMA CAR-mbIL-15 NK cells against hBCMA-NALM6-GFP-Luc target cells at indicated

effector-to-target (E:T) ratios.

Data are presented as mean ± SD from three independent healthy donor-derived cell preparations.

Statistical analysis was performed using the methods described in the main manuscript.

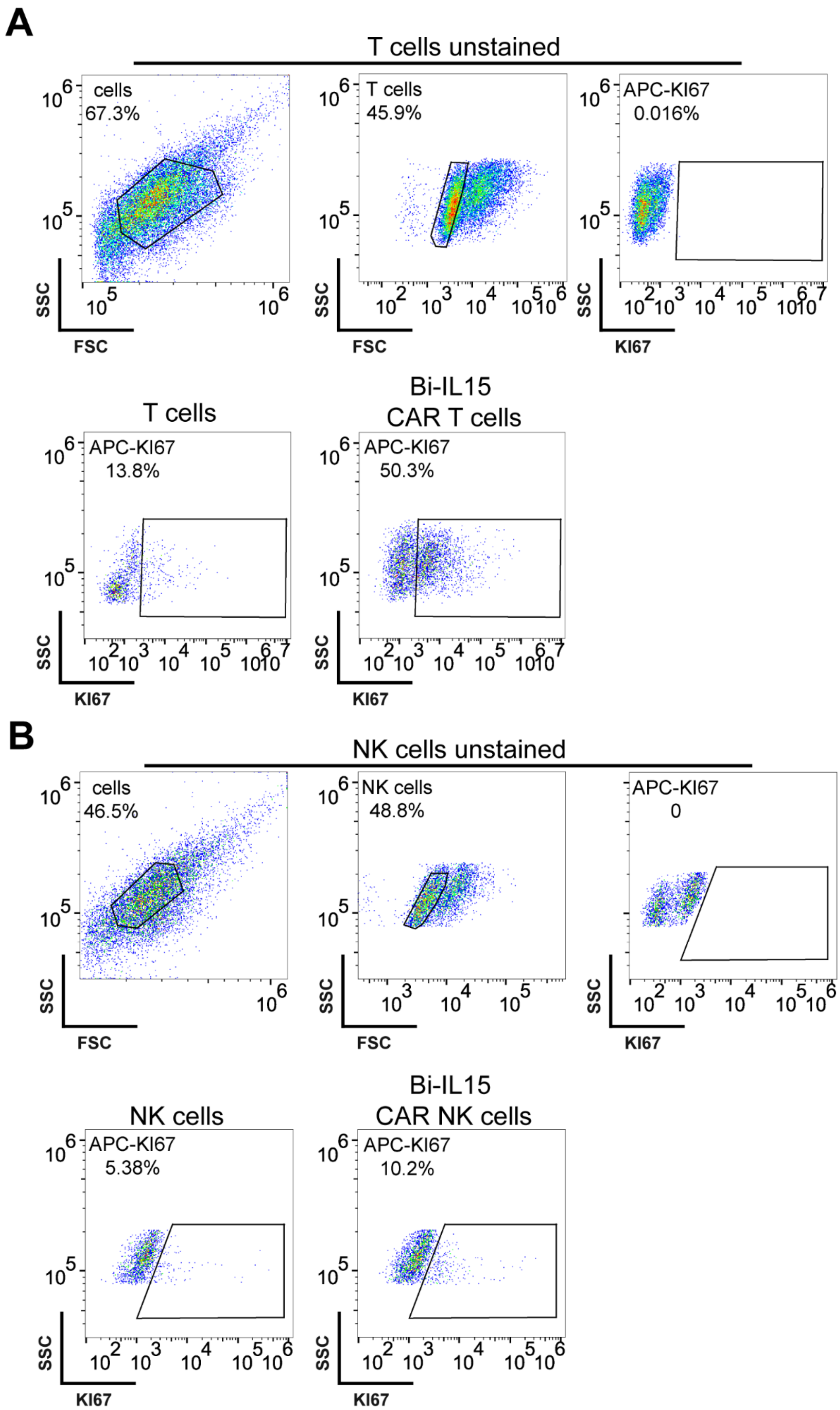

Supplementary Figure S5.

Representative flow cytometric analysis of Ki-67 expression in CAR-engineered T and NK

cells.

(A) Representative flow cytometric gating strategy and Ki-67 staining of untransduced T cells and bicistronic CD19/BCMA CAR-mbIL-15 T cells.

(B) Representative flow cytometric gating strategy and Ki-67 staining of untransduced NK cells and bicistronic CD19/BCMA CAR-mbIL-15 NK cells.

Unstained controls were used to define Ki-67-positive gates.

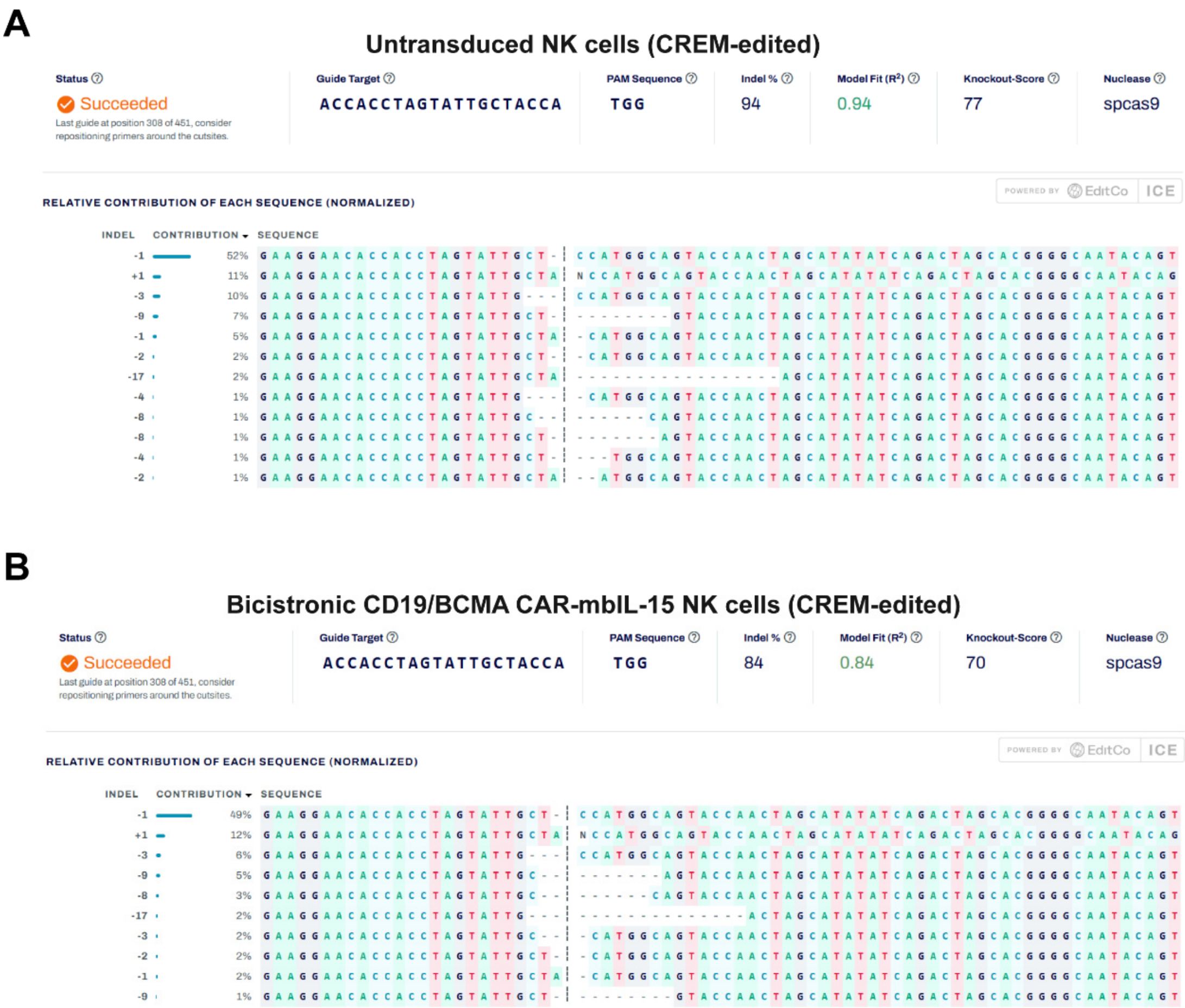

Supplementary Figure S6.

Validation of CREM editing by Sanger sequencing and ICE analysis.

(A) Representative Sanger sequencing trace and ICE analysis of the CREM target locus in CREM-edited NK cells.

(B) Representative Sanger sequencing trace and ICE analysis of the CREM target locus in CREM-edited bicistronic CD19/BCMA CAR-mbIL-15 NK cells.

Sanger sequencing and ICE analysis were performed using pooled genomic DNA samples generated

from three independent healthy donor-derived cell preparations.

Supplementary Table S1

**Product**	**Donor ID**	**Viability (%)**	**Phenotype (%)**	**CAR+(%)**	**VCN**
Bicistronic CD19/BCMA CAR-mbIL-15 NK	D1	92.7	$CD3^{-}CD56^{+}$ 98.4	73.2	4.7
Bicistronic CD19/BCMA CAR-mbIL-15 NK	D2	91.0	$CD3^{-}CD56^{+}$ 99.9	64.6	3.0
Bicistronic CD19/BCMA CAR-mbIL-15 NK	D3	94.2	$CD3^{-}CD56^{+}$ 99.8	71.4	4.5
Bicistronic CD19/BCMA CAR- mbIL-15 T	D1	92.0	$CD3^{+}$ 99.7	69.0	4.0
Bicistronic CD19/BCMA CAR- mbIL-15 T	D2	88.0	$CD3^{+}$ 99.9	65.0	3.6
Bicistronic CD19/BCMA CAR- mbIL-15 T	D3	94.0	$CD3^{+}$ 99.9	58.3	2.8

Donor information and characterization of representative bicistronic CD19/BCMA CAR-mbIL-15 NK and T-cell products.

Three independent healthy donor-derived PBMC preparations were used for generation of bicistronic CD19/BCMA CAR-mbIL-15 NK cells and CAR-mbIL-15 T cells. The table summarizes product characterization parameters, including cell viability, cellular phenotype, CAR expression, and vector copy number (VCN).
